# Does Surgical Resection Significantly Prolong the Long-Term Survival of Patients with Oligometastatic Pancreatic Ductal Adenocarcinoma? A Cross-Sectional Study Based on 18 Registries

**DOI:** 10.3390/jcm12020513

**Published:** 2023-01-08

**Authors:** Zheng Li, Xiaojie Zhang, Chongyuan Sun, Zefeng Li, He Fei, Dongbing Zhao

**Affiliations:** Department of Pancreatic and Gastric Surgical Oncology, National Cancer Center/National Clinical Research for Cancer/Cancer Hospital, Chinese Academy of Medical Sciences and Peking Union Medical College, Beijing 100021, China

**Keywords:** pancreatic ductal adenocarcinoma (PDAC), metastasis, surgical resection, oligometastatic pancreatic ductal adenocarcinoma, survival, prognosis

## Abstract

Background: Pancreatic ductal adenocarcinoma (PDAC) is a type of lethal gastrointestinal malignancy. It is mainly discovered at, and diagnosed with, an advanced stage of metastasis. As the only potentially curative treatment for PDAC, surgical resection has an uncertain impact on the survival of these patients. As such, we aimed to investigate if patients with metastatic PDAC (mPDAC) benefit from surgery. Methods: Patients with pancreatic cancer in 18 registries of the Surveillance, Epidemiology, and End Results database between 2000 and 2018 were reviewed retrospectively. According to the American Joint Committee on Cancer (AJCC), the eighth edition staging system was utilized. Propensity score matching was applied to strengthen the comparability of the study. The impact of surgery on survival was evaluated by restricted mean survival time (RMST) and Kaplan–Meier analysis. Results: A total of 210 well-matched mPDAC patients were included in the study. The 1 year, 3 year, and 5 year overall survival (OS) of patients undergoing surgery was 34.3%, 15.2%, and 11.0%, respectively. The 1 year, 3 year, and 5 year cancer-specific survival (CSS) of these patients was 36.1%, 19.7%, and 14.2%, respectively. RMST analysis revealed that mPDAC patients with surgery had better OS and CSS than those without (OS: 9.49 months vs. 6.45 months, *p* < 0.01; CSS: 9.76 months vs. 6.54 months, *p* < 0.01). Nevertheless, subgroup analysis demonstrated that such statistical significance especially existed in oligometastatic PDAC patients, which refers to those metastases that were limited in number and concentrated to a single organ in this study. Additionally, surgery was identified as a significant predictor for the long-term prognosis of patients (OS: [HR, hazard ratio] = 0.48, 95% CI: 0.36–0.65, *p* < 0.001; CSS: HR = 0.45, 95% CI: 0.33–0.63, *p* < 0.001). Lastly, a nomogram was established to predict whether an individual was suitable for surgical treatment in this study. Conclusions: Surgical resection significantly prolonged the long-term prognosis of oligometastatic PDAC patients. Such insights might broaden the management of patients with mPDAC to a large extent. However, a prospective clinical trial should be conducted before a recommendation of surgery in these patients.

## 1. Background

As the fourth leading cause of cancer-related mortality in the United States, pancreatic ductal adenocarcinoma (PDAC) has the worst clinical outcome among all cancer types, with a poor 5 year survival rate of approximately 11% [1,2]. To date, surgical resection remains the only potentially curative treatment for PDAC patients [3]. However, a majority of patients are asymptomatic and have distant metastases at the time of initial diagnosis, losing the chance to be surgical candidates according to the recent guidelines [1,4,5].

The NCCN Guidelines classify PDAC patients into several categories: resectable, borderline resectable, locally advanced unresectable (no metastases), and metastatic disease. For mPDAC management, further changes are needed as appropriate due to the specific status of patients. Clinical trial or chemotherapy is preferred for patients with good performance status, whereas single-agent chemotherapy or palliative radiation therapy is taken into consideration for those with poor performance status. However, surgical resection for mPDAC is not especially discussed in the NCCN Guidelines [5]. Considering the guidelines of different countries and regions, we further screened the European Society for Medical Oncology (ESMO) and International Study Group on Pancreatic Surgery (ISGPS) [6,7]. A gap in the surgical treatment of patients with mPDAC was found. In this setting, it is of significance to investigate whether patients with mPDAC can benefit from surgical resection.

With the remarkable progress achieved in surgical techniques and multidisciplinary consultation, primary resection of mPDAC patients can be carried out more broadly in high-volume centers after evaluation, which should be based on the utilization of appropriate imaging studies [5]. In the last few years, active attempts and related studies on primary surgery for patients with metastatic cancer of various types are gradually increasing, such as breast cancer [8,9], esophageal cancer [10], and colorectal cancer [11]. Concerning the various pathological subtypes of metastatic pancreatic cancer (PC), the surgical safety and feasibility give rise to a heated discussion [12,13,14,15,16]. However, certain limitations existed in these studies, and no consensus was reached.

Given the aforementioned considerations, we conducted this propensity-matched, cross-sectional, population-based Surveillance, Epidemiology, and End Results (SEER) analysis. The survival benefit of mPDAC patients receiving a surgical resection was evaluated using multiple analytical methods. Additionally, we further establish a nomogram to initially assess the criteria of a metastatic individual’s access to surgical resection in this study.

## 2. Methods

### 2.1. Patients and Study Design

**Figure 1:**
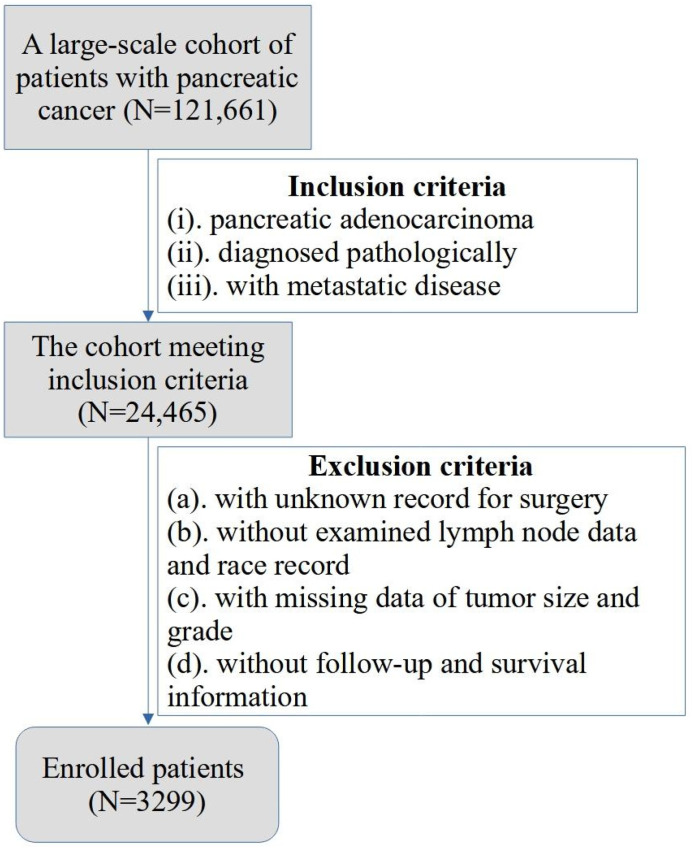
The flowchart for the process of patient screening.

The study included pathologically confirmed PC patients in the Surveillance, Epidemiology, and End Results database between 2000 and 2018. The “Incidence—SEER Research Plus Data, 18 Registries, Nov 2020 Sub (2000–2018)” database was selected, and the screening conditions were set as “positive histology and 8.6.4 carcinoma of pancreas”. Then, the clinicopathologic characteristics of 121,661 PC patients were obtained from the database, including sex, age, race record, year of diagnosis, tumor location, tumor size, tumor grade (differentiation), and details of tumor metastasis. Additionally, the staging of TNM was switched to the most recent eighth staging system according to the American Joint Committee on Cancer (AJCC). The inclusion criteria were as follows: (i) patients with PDAC; (ii) patients diagnosed pathologically; (iii) patients with metastatic disease. The exclusion criteria were as follows: (a) patients with unknown records for surgery; (b) patients with missing data of examined lymph node and race record; (c) patients without vital pathological information such as tumor size and grade; (d) patients who did not receive follow-up of their survival information. The process of patient screening is shown in a flowchart (Figure 1). As such, a total of 3299 patients were enrolled into the study. All the data were obtained and utilized under the data use agreement of SEER (ID: 16402-Nov2021).

### 2.2. Covariates and Outcomes

The covariates in this study mainly included four sections: (a) basic clinical characteristics: gender, age, race record, and years of diagnosis; (b) pathologic characteristics: tumor size, differentiation, tumor stage (AJCC 8th), details of tumor metastasis, examined lymph nodes, and positive lymph nodes; (c) treatment information: details of treatment, including surgery, chemotherapy, radiation, and systemic therapy; (d) follow-up data of survival: survival months, including overall survival (OS), and cancer-specific survival (CSS). All the data aforementioned were collected and provided by 18 registries affiliated with the National Cancer Institute (NCI).

### 2.3. Statistical Analysis

The analysis of all sorts in the study was performed with R software for Windows, Version 4.2.0. The chi-squared test or Fisher’s exact test was utilized to make an investigation in differences between categorical variables of the surgery group and non-surgery group, and the Mann–Whitney U test or t-test was used for comparing the differences of continuous variables between the two groups. Propensity score matching (PSM) was applied using R package “MatchIt” to equalize the unbalance between vital baseline characteristics of two groups, and the condition was set as “method = nearest, ratio = 1, caliper = 0.02”. The distribution of propensity scores was depicted in Supplementary Figure S1. We further evaluated the impact of surgery on long-term prognosis by restricted mean survival time (RMST) and Kaplan–Meier analysis (log-rank t-test) using R package “survRM2” and “survival”, respectively. The observation period for RMST was set at 20 months. Then, univariate and multivariate Cox proportional hazard regression models were established to determine the independent predictors of the prognosis of PDAC patients; all variables with *p* < 0.1 in univariate analysis were enrolled into multivariate analysis. Furthermore, on the basis of logistic regression analysis, a nomograph predicting whether a mPDAC patient will undergo surgery was constructed. All statistical analyses in this study were two-sided, and differences with *p* < 0.05 were considered statistically significant.

## 3. Results

### 3.1. Epidemiological Trend of mPDAC Patient Number

**Figure 2:**
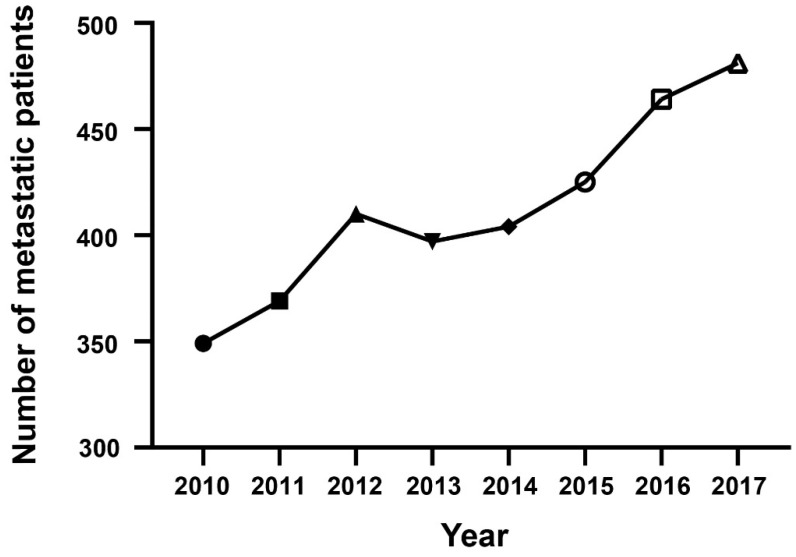
The epidemiological trend for the annual number of mPDAC patients.

A total of 3299 PDAC patients were enrolled into the study according to multiple inclusion and exclusion criteria. The number of patients with mPDAC was counted annually from 2010 to 2017, and the trend was depicted using a line chart (Figure 2). From the chart, an obvious conclusion can be drawn that the number of pathologically confirmed mPDAC patients had a significant upward trend, which made the already large disease burden more severe.

### 3.2. Baseline Characteristics

The baseline characteristics of mPDAC patients before and after PSM are summarized in Table 1. Before PSM, patients with mPDAC located at the pancreas head were more likely to receive a surgical resection (56.1% vs. 38.9%). Concerning treatment, chemotherapy (70.7%) and systemic therapy (58%) were performed in the majority of patients undergoing surgery. In the patients without surgery, 20.8% had no fewer than two metastatic organs, and liver metastasis (87% vs. 77.6%) and lung metastasis (22.3% vs. 13.7%) were more common than in patients with surgery. In terms of long-term prognosis, the survival of the surgery group was significantly better than that of the patients without surgery (mean [standard deviation, SD], 14.4 (16.7) months vs. 6.23 (8.11) months, *p* < 0.001). After one-to-one PSM, 105 pairs (surgery and non-surgery) of well-matched mPDAC patients were included in further analysis. The imbalance of baseline characteristics between the two groups was no longer statistically significant as expected. Nevertheless, the survival months of the surgery group were still significantly longer (mean [SD], 14.3 (18.9) months vs. 6.87 (8.33) months, *p* < 0.001).

### 3.3. Long-Term Survival Analysis

**Figure 3:**
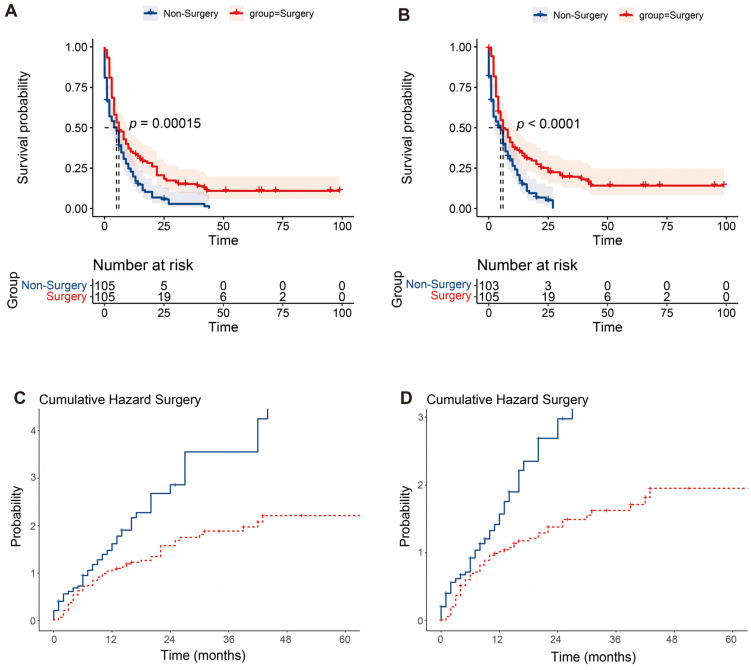
Survival analysis of no-surgery and surgery groups. The Kaplan–Meier analysis of overall survival (**A**) and cancer-specific survival (**B**) in two groups. Cumulative hazard concerning surgery of overall survival (**C**) and cancer-specific survival (**D**).

**Figure 4:**
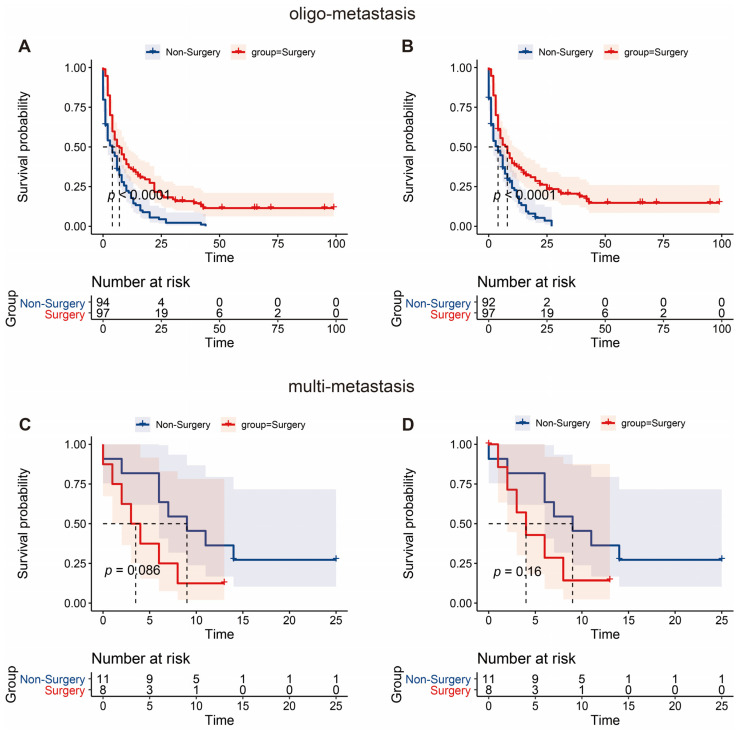
Survival analysis of patients with oligo- and multi-metastasis. The Kaplan–Meier analysis of overall survival (**A**) and cancer-specific survival (**B**) in PDAC patients with oligo-metastasis. Similar analysis of overall survival (**C**) and cancer-specific survival (**D**) in multi-metastatic PDAC patients.

**Figure 5:**
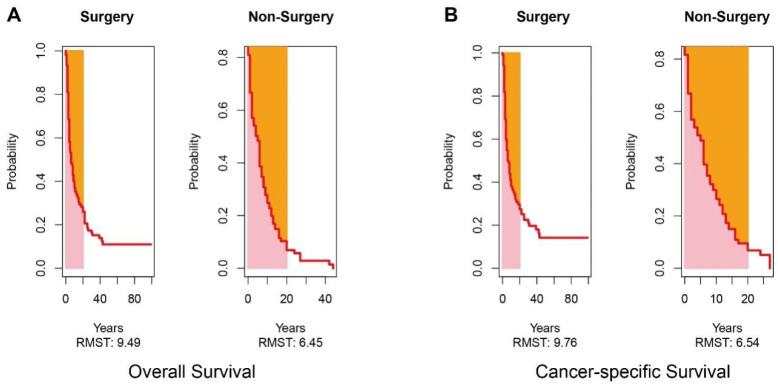
Analysis of restricted mean survival time (RMST). RMST analysis of overall survival (**A**) and cancer-specific survival (**B**) in two groups.

In the surgery group, the 1 year, 3 year, and 5 year OS of patients was 34.3%, 15.2%, and 11.0%, respectively. The 1 year, 3 year, and 5 year CSS of patients was 36.1%, 19.7%, and 14.2%, respectively. The Kaplan–Meier survival curve analysis demonstrated that the long-term survival of the surgery group was significantly better than that of the non-surgery group, including OS and CSS (all *p* < 0.001) (Figure 3). We further conducted such analysis for subgroups divided by the number of metastatic sites. However, statistically significant better OS and CSS were only observed in patients with one metastatic site; in simpler terms, oligometastatic mPDAC patients undergoing surgical section obtained better OS and CSS than those without surgery (all *p* < 0.001) (Figure 4). Additionally, since the distribution density of mPDAC patient survival mainly concentrated around 20 months, restricted mean survival time (RMST) of a period of 20 months was conducted to accurately evaluate the impact of surgery on the long-term prognosis of mPDAC patients. Compared to the non-surgery group, the RMST of patients undergoing surgery was significantly longer (OS: 9.49 months vs. 6.45 months, *p* < 0.01; CSS: 9.76 months vs. 6.54 months, *p* < 0.01) (Figure 5). Meanwhile, the restricted mean time lost (RMTL) was calculated, and the results are summarized in Table 2.

To further investigate the prognostic predictors of mPDAC patients in this study, univariate and multivariate Cox proportional hazard regression models were established. For OS, factors age, surgery, and chemotherapy were considered to have a significant impact on prognosis (all *p* < 0.05) (Table 3). Meanwhile, age, grade (differentiation), surgery, chemotherapy, and metastasis at distant lymph nodes were identified as independent prognostic factors in the survival analysis of CSS (all *p* < 0.05) (Table 4).

### 3.4. Development of a Nomogram Predicting Surgery

**Figure 6:**
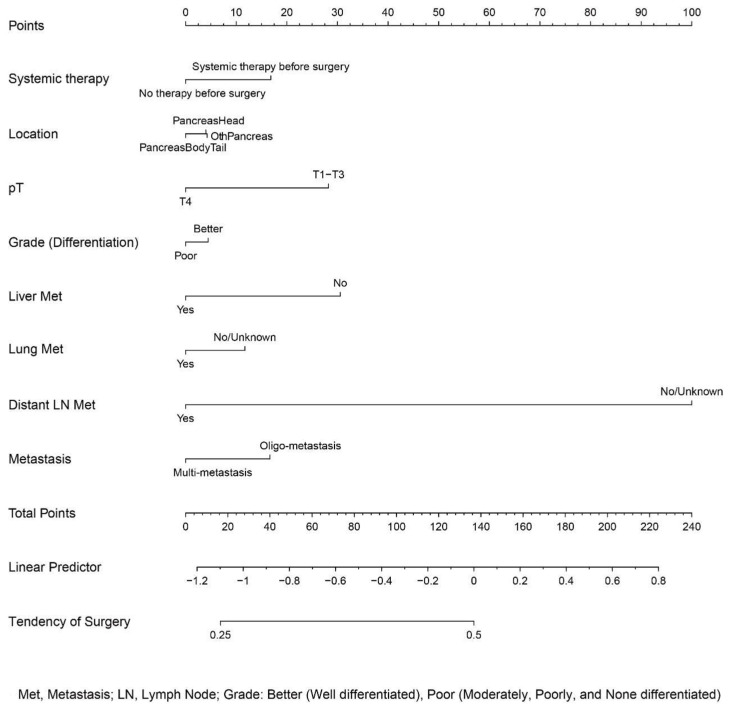
Nomogram for a preliminary prediction of surgery.

The patients provided with surgical resection were selected according to certain conditions. To determine the impact of surgery on the long-term prognosis of mPDAC patients, the prerequisites for a patient to receive the procedure should be determined. Accordingly, a nomogram predicting whether an individual received surgical treatment was constructed on the basis of logistic regression analysis. A series of metastatic, therapeutic, and pathological features were taken into consideration (Figure 6). By matching the characteristics and scores, we could approximately determine the probability of an individual obtaining the surgery.

## 4. Discussions

PDAC, a malignancy with a poor prognosis, is mainly diagnosed at an advanced, often metastatic stage [17]. According to multiple analytical methods in the current study, a significant prolonged long-term prognosis including overall survival (OS) and cancer-specific survival (CSS) was identified in mPDAC patients who underwent surgical resection, especially in PDAC patients with oligo metastasis. To the best of our knowledge, this study is the first to propose an investigation with such strictly matched control of clinicopathologic and therapeutic factors to determine the impact of surgical resection on long-term prognosis among patients with oligometastatic PDAC.

From the perspectives of NCCN guidelines [5], systemic therapy including radiotherapy and chemotherapy are considered options for the treatment of patients with mPDAC. Nevertheless, radiotherapy is only performed for palliative purposes in patients with metastatic disease progression, while the options of first-line chemotherapy are FOLFIRINOX and gemcitabine regimens for patients with good and poor performance status, respectively. Regarding the outcome of these patients, although prolonged survival was obtained after chemotherapy treatment only, the development of new chemotherapeutic agents was stagnant for a long time, thus restraining the improvement of long-term survival in these patients. Moreover, there were no encouraging clinical benefits observed in the presence of molecule-targeted drugs, including cetuximab, bevacizumab, and axitinib [18,19,20]. In this setting, views should be converted to explore more options for treatment for metastatic patients with PDAC.

Surgical resection was considered the best potential curative therapy for non-metastasis patients with PDAC but not indicated for mPDAC patients due to the consideration of limited safety and efficacy in the past decades. As a matter of fact, with noticeable progress in surgical techniques and procedures, extended surgical approaches can also be carried out with low morbidity and mortality rates in well-selected metastatic patients [21]. Accumulating evidence has provided support for this view. The population-based study of Hamad et al. divided 47,785 mPDAC patients with liver-only metastasis into a nonsurgical group (*n* = 46,894) and a surgical group (*n* = 891). The study demonstrated that the median overall survival of the surgical group was significantly higher compared to the nonsurgical group (10.74 months vs. 3.4 months *p* < 0.001), suggesting that surgery was associated with improved survival of well-selected PDAC patients with liver metastasis [13]. Younghwan et al. conducted a study including 70 stage IV PDAC patients (palliative pancreatic resection group, *n* = 35 and bypass or biopsy group, *n* = 35), which were matched according to tumor size and peritoneal seeding. A conclusion was drawn from the study that resection for PDAC patients stage IV can be associated with prolonged survival [22]. Yang et al. focused on synchronous resection inpatients with liver metastatic PDAC located at the pancreas body/tail. A better OS was identified in patients who underwent resection than those who did not (16.1 months vs. 6.4 months, *p* = 0.02) [23].

However, certain limitations also existed in these previous studies. In addition to being retrospective studies, the absence of matching control of systemic treatment sequences in these studies can lead to compromised results. Systemic therapy before surgical resection could improve the clinicopathologic characteristics of these metastatic patients. Approximately one-third of initially staged nonresectable PDAC patients would be converted to resectable following neoadjuvant therapy, with comparable survival to initially resectable patients [24]. Moreover, after surgery, chemotherapy has also been proven to be a vital supportive treatment with respect to both survival and quality of life [17]. In brief, the therapeutic sequence of systematic treatment around surgical resection should be especially considered owing to its great impact on prognosis. Given the aforementioned considerations, we classified the sequence of systemic therapy into before surgery, after surgery, both before and after surgery, and no systemic therapy. Then, PSM was performed for a strictly matched control in patients from the surgery group and non-surgery group.

As a matter of fact, the patients in this study treated with surgical resection were well selected according to certain conditions. When discussing the impact of surgery on long-term prognosis of mPDAC patients, the prerequisites for a patient to receive the procedure should be clear. At the end of the study, a nomogram predicting whether an individual should be offered a surgical resection was established on the basis of logistic regression analysis. A series of metastatic, therapeutic, and pathological features were taken into consideration in the model. To the best of our knowledge, the current study is the first to further investigate the standards for an individual to receive surgery. However, the nomogram is only able to make a preliminary prediction on whether the patient is suitable for surgical resection, and a consensus based on clinical trials should be established in the future.

Certainly, the limitations of the present study should be acknowledged. Firstly, this was a cross-sectional study, and biases existed because of its nature. Secondly, due to the limited information from the Surveillance, Epidemiology, and End Results database, we could not access the details of chemotherapy regimens. Therefore, although relatively sufficient matching control was performed, a small amount of bias due to different therapeutic regimens still existed. In addition, the registries of SEER did not provide data on patient performance status, the volume of the tumor, or location of the metastases. According to previous studies, perioperative mortality can be as low as 0%, regarding resection of PC combined with synchronous metastasectomy [25]. Pancreaticoduodenectomy (PD) with synchronous liver metastasectomy for oligometastatic PDAC is safe and feasible, and it might provide survival benefits for selected patients [26]. Regarding the M1 periampullary cancer of the pancreas, pancreatic resection together with metastasectomy can be performed safely in well-selected patients [27]. In this study, surgical resection was also observed to significantly prolong the survival of oligometastatic PDAC patients. However, the information on whether the patients followed metastaseconomy was uncertain, and the factor of liver metastasis was eliminated by PSM analysis, which provided us room for further improvement in this study. Additionally, the surgery of mPDAC patients should be performed with safety, feasibility, and ethical rationality. The validity of the treatment was only assessed by prolonged survival of mPDAC patients due to the lack of the status of surgical margin and other important factors, leading to a limited conclusion on surgical validity. Therefore, even if the surgical resection can bring prolonged survival of patients with mPDAC in this study, such surgery should not be recommended before its safety and feasibility are confirmed by clinical trials. Thirdly, the number of PDAC patients with multi-metastases in the analysis was relatively low, which could have compromised the results. Nevertheless, the findings of this study provide new and useful insights into the clinical management of patients with mPDAC.

## 5. Conclusions

The favorable impact of surgical resection on the prognosis of oligometastatic PDAC patients was well recognized in the study. The long-term OS and CSS were significantly prolonged according to multiple strictly adjusted analyses. Nevertheless, there is still room for further research, such as whether patients’ quality of life benefits from palliative surgery. We did not conduct this study to recommend surgical resection for oligometastatic PDAC patients; prospective, randomized clinical trials are still needed to provide reliable support in the future.

## Figures and Tables

**Table 1:** Baseline characteristics of mPDAC patients before and after propensity score matching.

Characteristics	Raw Data of mPDAC Patients			Data after Propensity Score Matching		
	Non-Surgery (*n* = 3094)	Surgery (*n* = 205)	*p*-Value	Non-surgery (*n* = 105)	Surgery (*n* = 105)	*p*-Value
Sex, *n* (%)						
Female	1383 (44.7%)	96 (46.8%)	0.838	52 (49.5%)	48 (45.7%)	0.858
Male	1711 (55.3%)	109 (53.2%)		53 (50.5%)	57 (54.3%)	
Age, *n* (%)						
<65	1216 (39.3%)	80 (39.0%)	0.065	44 (41.9%)	41 (39.0%)	0.974
≥85	190 (6.1%)	1 (0.5%)		32 (30.5%)	35 (33.3%)	
65–74	992 (32.1%)	74 (36.1%)		29 (27.6%)	28 (26.7%)	
75–84	696 (22.5%)	50 (24.4%)		0 (0%)	1 (1.0%)	
Race, *n* (%)						
Black	401 (13.0%)	17 (8.3%)	0.436	9 (8.6%)	12 (11.4%)	0.789
Other (American Indian/AK Native, Asian/Pacific Islander)	260 (8.4%)	18 (8.8%)		6 (5.7%)	10 (9.5%)	
White	2433 (78.6%)	170 (82.9%)		90 (85.7%)	83 (79.0%)	
Location, *n* (%)						
OthPancreas	663 (21.4%)	25 (12.2%)	<0.001	18 (17.1%)	18 (17.1%)	1
PancreasBodyTail	1228 (39.7%)	65 (31.7%)		25 (23.8%)	25 (23.8%)	
PancreasHead	1203 (38.9%)	115 (56.1%)		62 (59.0%)	62 (59.0%)	
Grade, *n* (%)						
Moderately differentiated; Grade II	1243 (40.2%)	71 (34.6%)	0.634	43 (41.0%)	38 (36.2%)	0.997
Poorly differentiated; Grade III	1617 (52.3%)	122 (59.5%)		54 (51.4%)	59 (56.2%)	
Undifferentiated; anaplastic; Grade IV	52 (1.7%)	2 (1.0%)		2 (1.9%)	2 (1.9%)	
Well differentiated; Grade I	182 (5.9%)	10 (4.9%)		6 (5.7%)	6 (5.7%)	
AJCC 8th pT, *n* (%)						
T1	50 (1.6%)	4 (2.0%)	0.802	1 (1.0%)	1 (1.0%)	0.975
T2	299 (9.7%)	27 (13.2%)		13 (12.4%)	12 (11.4%)	
T3	315 (10.2%)	22 (10.7%)		5 (4.8%)	9 (8.6%)	
T4	2430 (78.5%)	152 (74.1%)		86 (81.9%)	83 (79.0%)	
Radiotherapy, *n* (%)						
None/unknown	2936 (94.9%)	189 (92.2%)	0.246	102 (97.1%)	98 (93.3%)	0.432
Yes	158 (5.1%)	16 (7.8%)		3 (2.9%)	7 (6.7%)	
Chemotherapy, *n* (%)						
No/unknown	1306 (42.2%)	60 (29.3%)	0.001	59 (56.2%)	58 (55.2%)	0.99
Yes	1788 (57.8%)	145 (70.7%)		46 (43.8%)	47 (44.8%)	
Systemic therapy, *n* (%)						
No systemic therapy	3023 (97.7%)	60 (29.3%)	<0.001	59 (56.2%)	58 (55.2%)	0.999
Systemic therapy after surgery	54 (1.7%)	119 (58.0%)		37 (35.2%)	36 (34.3%)	
Systemic therapy before surgery	12 (0.4%)	14 (6.8%)		6 (5.7%)	8 (7.6%)	
Systemic therapy both before and after surgery	5 (0.2%)	12 (5.9%)		3 (2.9%)	3 (2.9%)	
Bone metastasis, *n* (%)						
NA	77 (2.5%)	5 (2.4%)	0.997	2 (1.9%)	3 (2.9%)	0.987
No	2847 (92.0%)	190 (92.7%)		99 (94.3%)	99 (94.3%)	
Yes	170 (5.5%)	10 (4.9%)		4 (3.8%)	3 (2.9%)	
Brain metastasis, *n* (%)						
NA	74 (2.4%)	5 (2.4%)	0.856	2 (1.9%)	3 (2.9%)	0.903
No	3000 (97.0%)	200 (97.6%)		103 (98.1%)	102 (97.1%)	
Yes	20 (0.6%)	0 (0%)				
Liver metastasis, *n* (%)						
NA	14 (0.5%)	0 (0%)	0.002	16 (15.2%)	18 (17.1%)	0.932
No	387 (12.5%)	46 (22.4%)		89 (84.8%)	87 (82.9%)	
Yes	2693 (87.0%)	159 (77.6%)				
Lung metastasis, *n* (%)						
NA	86 (2.8%)	3 (1.5%)	0.036	2 (1.9%)	2 (1.9%)	1
No	2319 (75.0%)	174 (84.9%)		90 (85.7%)	91 (86.7%)	
Yes	689 (22.3%)	28 (13.7%)		13 (12.4%)	12 (11.4%)	
Distant lymph node metastasis, *n* (%)						
NA	2224 (71.9%)	142 (69.3%)	0.731	84 (80.0%)	78 (74.3%)	0.509
No	761 (24.6%)	52 (25.4%)		15 (14.3%)	24 (22.9%)	
Yes	109 (3.5%)	11 (5.4%)		6 (5.7%)	3 (2.9%)	
Other metastasis, *n* (%)						
NA	2227 (72.0%)	142 (69.3%)	0.236	84 (80.0%)	78 (74.3%)	0.83
No	678 (21.9%)	42 (20.5%)		16 (15.2%)	18 (17.1%)	
Yes	189 (6.1%)	21 (10.2%)		5 (4.8%)	9 (8.6%)	
Metastasis, *n* (%)						
Multi-metastasis	644 (20.8%)	22 (10.7%)	0.002	11 (10.5%)	8 (7.6%)	0.771
Oligo-metastasis	2450 (79.2%)	183 (89.3%)		94 (89.5%)	97 (92.4%)	
Number of regional nodes examined						
Mean (SD)	NA	14.7 (12.4)		NA	14.3 (12.0)	
Median [min, max]	NA	13.0 [0, 84.0]		NA	13.0 [0, 68.0]	
Number of regional nodes positive						
Mean (SD)	NA	3.21 (4.26)		NA	3.10 (3.71)	
Median [min, max]	NA	2.00 [0, 20.0]		NA	2.00 [0, 14.0]	
Size						
Mean (SD)	46.2 (32.3)	44.0 (25.1)	0.49	43.0 (20.7)	45.2 (25.5)	0.774
Median [min, max]	42.0 [0, 900]	39.0 [0, 188]		40.0 [0, 103]	40.0 [0, 185]	
Survival months						
Mean (SD)	6.23 (8.11)	14.4 (16.7)	<0.001	6.87 (8.33)	14.3 (18.9)	<0.001
Median [min, max]	3.00 [0, 82.0]	9.00 [0, 99.0]		4.00 [0, 44.0]	6.00 [0, 99.0]	

**Table 2:** RMST and RMTL analysis for mPDAC patients.

Analysis	Overall Survival			Cancer-Specific Survival		
	Non-Surgery	Surgery	*p*-Value	Non-Surgery	Surgery	*p*-Value
	Months (95%CI)	Months (95%CI)		Months (95%CI)	Months (95%CI)	
Restricted mean survival time	6.453 (5.198–7.709)	9.489 (8.062–10.916)	<0.01	6.54 (5.254–7.826)	9.76 (8.311–11.209)	<0.01
Restricted mean time lost	13.547 (12.291–14.802)	10.511 (9.084–11.938)	<0.01	13.46 (12.174–14.746)	10.24 (12.174–14.746)	<0.01

**Table 3:** Cox regression analysis of overall survival.

Characteristics	Univariate Analysis		Multivariate Analysis	
	HR (95%CI)	*p*-Value	HR (95%CI)	*p*-Value
Sex (male vs. female)	0.76 (0.57–1.01)	0.057	0.79 (0.58–1.07)	0.126
Age (reference <65)				
≥85	0.67 (0.09–4.86)	0.695	1.22 (0.16–9.15)	0.849
65–74	1.18 (0.84–1.67)	0.334	1.17 (0.82–1.66)	0.376
75–84	2.6 (1.82–3.71)	<0.001	2.07 (1.41–3.05)	<0.001
Race (reference Black)				
Other	0.78 (0.39–1.57)	0.492		
White	1.06 (0.66–1.7)	0.823		
Location (reference OthPancreas)				
PancreasBodyTail	1.32 (0.83–2.09)	0.243		
PancreasHead	1.35 (0.9–2.03)	0.142		
Grade (reference Grade II)				
Grade III	1.54 (1.14–2.09)	0.005	1.3 4(0.97–1.85)	0.08
Grade IV	2.43 (0.88–6.7)	0.086	1.69 (0.6–4.8)	0.324
Grade I	0.94 (0.47–1.89)	0.867	0.52 (0.26–1.08)	0.079
pT (reference T1)				
T2	0.57 (0.13–2.44)	0.45		
T3	0.72 (0.16–3.22)	0.668		
T4	0.77 (0.19–3.1)	0.708		
Surgery (yes vs. No)	0.58 (0.43–0.77)	<0.001	0.48 (0.36–0.65)	<0.001
Radiotherapy (yes vs. No)	0.6 (0.31–1.18)	0.137		
Chemotherapy (yes vs. No)	0.35 (0.26–0.47)	<0.001	0.37 (0.26–0.52)	<0.001
Systemic therapy (yes vs. No)	0.38 (0.22–0.65)	<0.001	0.71 (0.4–1.28)	0.259
Bone metastasis (reference NA)				
No	0.54 (0.22–1.31)	0.172		
Yes	0.56 (0.17–1.85)	0.343		
Brain metastasis (reference NA)				
No	0.49 (0.2–1.19)	0.114		
Liver metastasis (reference NA)				
No	0.98 (0.67–1.44)	0.921		
Lung metastasis (reference NA)				
No	0.67 (0.25–1.82)	0.431		
Yes	1.05 (0.37–3.03)	0.923		
Distant lymph node metastasis (reference NA)				
No	0.78 (0.53–1.14)	0.195		
Yes	0.63 (0.3–1.35)	0.237		
Other metastasis (reference NA)				
No	0.79 (0.53–1.19)	0.266		
Yes	0.64 (0.35–1.19)	0.16		
Metastasis (oligo- vs. multi-)	1.07 (0.63–1.81)	0.809		

**Table 4:** Cox regression analysis of cancer-specific survival.

Characteristics	Univariate Analysis		Multivariate Analysis	
	HR (95%CI)	*p*-Value	HR (95%CI)	*p*-Value
Sex (male vs. female)	0.77 (0.57–1.04)	0.093	0.8(0.58–1.1)	0.173
Age (reference <65)				
≥85	0 (0–Inf)	0.995	0 (0–Inf)	0.993
65–74	1.2 (0.84–1.72)	0.32	1.18 (0.81–1.72)	0.399
75–84	2.55 (1.77–3.69)	<0.001	1.99 (1.33–3)	<0.001
Race (reference Black)				
Other	0.8 (0.39–1.65)	0.548		
White	1.12 (0.68–1.86)	0.649		
Location (reference OthPancreas)				
PancreasBodyTail	1.4 (0.86–2.28)	0.177	1.66 (0.98–2.81)	0.058
PancreasHead	1.5 (0.97–2.31)	0.067	1.47 (0.93–2.34)	0.1
Grade (reference Grade II)				
Grade III	1.74 (1.27–2.41)	0.001	1.54 (1.1–2.17)	0.013
Grade IV	1.99 (0.62–6.37)	0.247	1.37 (0.42–4.52)	0.605
Grade I	1.07 (0.53–2.15)	0.853	0.74 (0.35–1.57)	0.434
pT (reference T1)				
T2	0.48 (0.11–2.1)	0.332		
T3	0.66 (0.15–2.96)	0.583		
T4	0.75 (0.19–3.05)	0.691		
Surgery (yes vs. No)	0.54 (0.4–0.73)	<0.001	0.45 (0.33–0.63)	<0.001
Radiotherapy (yes vs. No)	0.64 (0.33–1.25)	0.193		
Chemotherapy (yes vs. No)	0.33 (0.24–0.45)	<0.001	0.38 (0.27–0.55)	<0.001
Systemic therapy (yes vs. No)	0.35 (0.2–0.63)	<0.001	0.67 (0.35–1.25)	0.206
Bone metastasis (reference NA)				
No	0.52 (0.21–1.27)	0.15		
Yes	0.47 (0.14–1.63)	0.233		
Brain metastasis (reference NA)				
No	0.47 (0.19–1.14)	0.095	0.5 (0.19–1.34)	0.169
Liver metastasis (reference NA)				
No	1.01 (0.68–1.51)	0.945		
Lung metastasis (reference NA)				
No	0.64 (0.24–1.75)	0.388		
Yes	1 (0.35–2.9)	0.994		
Distant lymph node metastasis (reference NA)				
No	0.69 (0.46–1.04)	0.075	0.81 (0.53–1.25)	0.352
Yes	0.65 (0.3–1.38)	0.26	0.44 (0.2–0.97)	0.041
Other metastasis (reference NA)				
No	0.72 (0.47–1.11)	0.135		
Yes	0.6 (0.31–1.13)	0.115		
Metastasis (oligo- vs. multi-)	1.1 (0.64–1.91)	0.723		

## Data Availability

The datasets used during the current study are available from the corresponding author on reasonable request.

## Supplementary Materials

The following supporting information can be downloaded at https://www.mdpi.com/article/10.3390/jcm12020513/s1: Figure S1. Propensity score matching (PSM). Distribution of propensity scores (A) and proportion of propensity score before and after matching (B).
