# Supplementary material for: Does Surgical Resection Significantly Prolong the Long-Term Survival of Patients with Oligometastatic Pancreatic Ductal Adenocarcinoma? A Cross-Sectional Study Based on 18 Registries
Source: J Clin Med. 2023 Jan 8;12(2):513. doi: 10.3390/jcm12020513 (PMC9867229; doi:10.3390/jcm12020513)
Supplement: Supplementary file 1 [file jcm-12-00513-s001.zip › jcm-2047679-supplementary.pdf]

Supplementary Figure

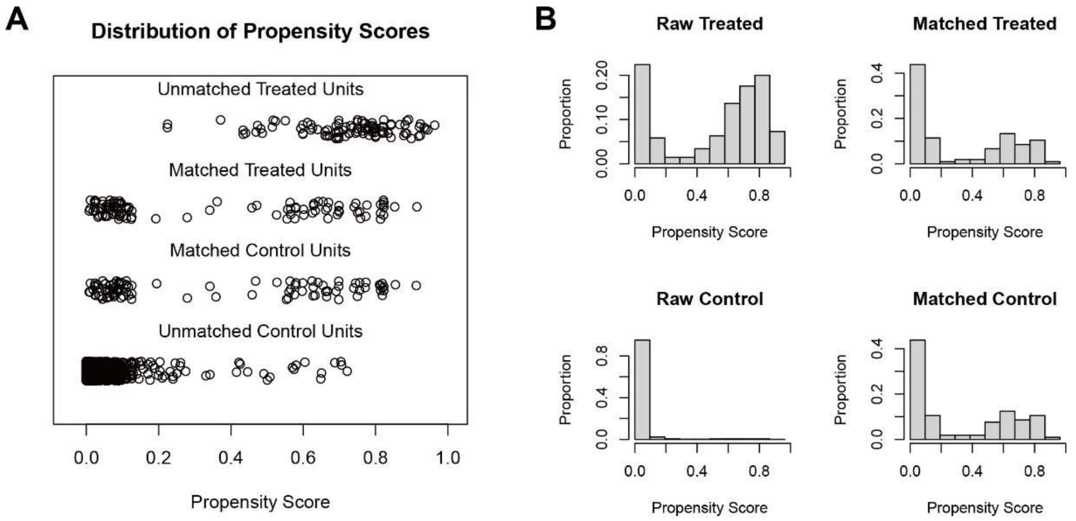

Figure S1. Propensity score matching (PSM). Distribution of propensity scores (A) and proportion of propensity score before and after matching (B).
